# Annotations

**Published:** 1905-10-07

**Authors:** 


### ANNOTATIONS.

### The Encouragement of Research.

One of the most interesting introductory addresses at the opening of the Medical Schools on Monday was delivered at St. George's Hospital by Mr. Brudenell Carter. After referring to medical education, and the importance of physics, Mr. Carter dwelt at some length on the need of research work in hospitals, and pleaded for its encouragement, insisting that endowments and donations for researches likely to lead to the preservation of health and life, and to the diminution of suffering, are just as essential as they are for the sustentation of the patients in the wards; he contrasted the readiness with which the public contribute to the one with the indisposition they manifest in rendering assistance to the other. Yet, as Mr. Carter pointed out, in the former case a sick man and his family may benefit, while in the latter the welfare of the human race may be at stake. Why does this indifference prevail? Mr. Carter explains it on the ground that the public do not understand the question which is at issue, and we think that he is not far wide of the mark. It is not always easy to induce the public to provide money for a scheme which they understand—it is impossible to extract it from them for a project which they cannot master. The moral is that those who are acquainted with the question at issue and know how much hangs upon it, should use every opportunity which offers to show in the clearest possible manner the practical advantages accruing to the community from the endowment of scientific research.

### The Tuberculosis Congress.

Judged by the general interest which it has aroused, and by the large number of eminent and representative medical men of different countries who are attending it, the Congress on Tuberculosis, which is being held in Paris this week, must be regarded as a great success. On Tuesday, when the real work of the Congress began, papers were read upon the relationship between human and bovine tuberculosis, constitutional and hereditary disposition and the means of modifying them, the future possibilities of serum therapy, preservation of infant life, isolation of tuberculous patients, and insurance in its connection with the prevention of consumption among the working classes. It is true that no epoch-making announcement was forthcoming upon any of these points, nevertheless, by reflecting the most authoritative medical opinions of to-day and attracting public notice towards the subjects discussed a good deal of popular enlightenment is likely to be the immediate outcome of the Congress. And after all, the hygienic education of the laity is perhaps the chief means at present available for the defence of mankind against the bacillus of tuberculosis. In the discussion on human and bovine tuberculosis it was apparent that the vast majority of medical opinion was directly opposed to Koch's view that the causative organisms belong to distinct species; and the following resolution was passed almost unanimously:—"That the Congress, after hearing the *exposé* of the most recent investigations, declares that it is not only indispensable to avoid contagion from man to man, but also to pursue the prophylaxis of bovine tuberculosis and to continue to take administrative and hygienic measures to avert its possible transmission to our species, and finally that it is desirable to be on our guard against all forms of animal tuberculosis." It is true that the Royal Commission appointed in 1901 in inquire into the relations of human and animal tuberculosis had already practically decided the question so far as this country is concerned, in favour of Professor Koch's opponents, but it is none the less satisfactory to observe comparative unanimity on such an important national and international question among the representatives of nearly every civilised country to-day.

### A Failure of Duty.

The correspondence which has been published this week between the Guardians of Southwark Union and the authorities of Guy's Hospital not only effectually disposes of certain specific allegations recently made against the latter, but brings to the front a question of great and pressing importance. The allegations had reference to a woman residing in the borough, who on July 11 was sent away from Guy's Hospital to the relief station in the Borough Road belonging to the Southwark Guardians; and as she arrived there in a condition declared by the relieving officer to be very critical, the Clerk to the Guardians was instructed to inquire why she was not admitted at Guy's. The Treasurer of Guy's, under date of September 28, supplies this information. He states that when the patient was brought to Guy's Hospital by her husband on July 11, her serious condition was recognised, but she could not then be admitted because there was not a single female medical bed available. The medical officers, however, considering that it was a case which should forthwith have the advantage of better medical and nursing attendance than she could obtain at home, sent her in a cab, at the expense of the hospital, to the relief station. The Treasurer, very properly, not content to rebut the implication of "a scandal at Guy's," expressed on behalf of the Governors, as large ratepayers, their regret that the Southwark Guardians do not provide infirmary accommodation within the limits of their Union. This is the crux of the whole matter. Dr. Paton, the district medical officer, ordered the woman to be removed from the relief station to St. George's Workhouse "because she would very likely have died on the journey to the infirmary at Dulwich." We agree that St. George's Workhouse is not at all a suitable place for a person who is dangerously ill. But if the patient had been sent to Dulwich and had died on the way, the responsibility for her death would have rested at the door of the Southwark Guardians. It is all very well to spend money lavishly on an infirmary four miles distant; but the Guardians are guilty of a serious failure of duty so long as they refrain from supplying adequate accommodation for the sick poor in their midst, and the authorities of Guy's have read them a lesson which we hope they will take to heart.

